# Do Professions Remain Analytically Distinct From Non‐Professional Occupations?

**DOI:** 10.1111/cars.70056

**Published:** 2026-09-28

**Authors:** Meaghan Furlano

**Affiliations:** ^1^ Department of Sociology Western University London Canada

**Keywords:** attacks on professions, occupations, precariousness, professions, sociology of work

## Abstract

Recent debates in sociological literature have questioned whether ‘professions’ and ‘non‐professional occupations’ remain analytically distinct concepts. This article engages with longstanding scholarship on work, occupations and professions to address this question. Drawing primarily on the work of Adams (2018), Saks (2021) and their associated neo‐Weberian approaches, I argue that professions are socially constructed and historically, geographically and temporally contingent. As such, the contemporary macrostructural conditions (re)shaping professional work in the Anglo‐American context—most notably emerging subjective forms of precariousness—should not be interpreted as signalling the demise of professions. Rather, they represent the latest iteration of professional work and its ongoing effort to maintain hegemony amid shifting economic structures. Although professional and non‐professional occupations appear to be converging in some of the respects traditionally separating them, I argue that professions remain analytically distinct from non‐professions. This distinction persists because professionals continue to benefit from the economic and symbolic advantages they have secured through state legitimation, social closure, and the enduring cultural representations of themselves and their work that promote the ideological ‘common‐senseness’ of their elevated social and economic status. The article therefore concludes that professions are not experiencing a ‘crisis’ but are instead entering a new phase of their ongoing reconstruction.

## RÉSUMÉ

Des débats récents dans la littérature sociologique ont remis en question le fait que les « professions » et les « métiers non professionnels » constituent encore des concepts distincts sur le plan analytique. Cet article s'appuie sur une longue tradition de recherches consacrées au travail, aux métiers et aux professions pour aborder cette question. En m'appuyant principalement sur les travaux d'Adams (2018) et de Saks (2021), ainsi que sur leurs approches néo‐wébériennes, je soutiens que les professions sont des constructions sociales et qu'elles sont contingentes sur les plans historique, géographique et temporel. À ce titre, les conditions macrostructurelles contemporaines qui (re)façonnent le travail professionnel dans le contexte anglo‐américain — notamment les formes subjectives émergentes de précarité — ne doivent pas être interprétées comme annonçant la disparition des professions. Elles représentent plutôt la dernière itération du travail professionnel et son effort continu pour maintenir son hégémonie au milieu de structures économiques en mutation. Bien que les professions et les métiers non professionnels semblent converger sur certains des aspects qui les séparaient traditionnellement, je soutiens que les professions restent analytiquement distinctes des non‐professions. Cette distinction persiste car les professionnels continuent de bénéficier des avantages économiques et symboliques qu'ils se sont assurés grâce à la légitimation de l’État, à la fermeture sociale et aux représentations culturelles durables d'eux‐mêmes et de leur travail, qui promeuvent le « bon sens » idéologique de leur statut social et économique élevé. L'article conclut donc que les professions ne traversent pas une « crise », mais entrent au contraire dans une nouvelle phase de leur reconstruction continue.

## 1 Introduction

In this article, I engage with the lively debate about whether professions remain analytically distinct from non‐professional occupations and, if so, how scholars should best define them. Exploring their ‘analytic distinction’ means conceptually separating out—or ‘pulling apart’—‘professions’ and ‘non‐professions’ for the purposes of studying them more clearly. That can include studying their differences, but also their areas of overlap. Pulling together insights from various literatures, I trace the emergence of professional work and analyze the transformations currently (re)shaping it in Anglo‐American contexts, with a special focus on the Canadian context owing to this journal's audience and interests. Doing so enables me to track which of the characteristics associated with early professional work remain prominent. It also leads me to consider how new forms of social closure are being enacted to protect professional status against interlopers (e.g., Faulconbridge et al. 2023; see Abbott 1988).

I draw heavily on Adams (2018), Saks (2021) and their associated neo‐Weberian approaches to argue that because professions are social constructs that are historically, geographically and temporally amenable (see Adams 2010, 2015), there cannot be a universal, static definition of professions. This is not, however, to suggest that professions are undefinable. As Adams (2010, 65) notes: ‘if one examines which occupations have status in certain locations and at certain points in time, one should be able to identify professions’, usually on account of their esteemed legal, social and cultural status. I should note at the outset of this article that I am primarily guided by, and will be engaging mostly with, the neo‐Weberian approach to analyzing professions (see Adams and Saks 2018; Saks 1983, 2010, 2012, 2021; Saks and Adams 2019). In light of Saks’ (2010, 887) persuasive demonstration that the issues posed by other approaches (e.g., interactionist, functionalist) are ‘largely addressed by the neo‐Weberian approach’, I am apt to agree that ‘the neo‐Weberian approach remains the most incisive and empirically fruitful perspective on the sociology of professions’, particularly in advanced Anglo‐American societies (see Saks 2010, for a more exhaustive account).

I also recognize that while all professions are occupations, not all occupations are professions. Hence, professions are recognizable through the distinctive features that separate them from ‘other’ occupations. As Adams (2018, 19–20, emphasis added) argues, ‘Professions are *occupational status groups*: workers who have more social status, and a different legal status than other workers’. These ‘other’ occupations, henceforth referred to as ‘non‐professions’, or ‘non‐professional occupations’, are defined as the jobs and activities persons undertake to earn a living. They tend to require comparatively less human capital, have lower standards to entry, and are not subject to strict ethical codes. Importantly, non‐professional occupations have not yet achieved the professional ‘success’ of excluding outsiders or securing higher income, status and power (Saks 2010), though not necessarily for lack of trying (Adams 2018).

In agreement with Adams (2018), I contend that the most robust definition of professions acknowledges their variability whilst also emphasizing the persistence of certain core dimensions across social contexts (Abbott 1988; Adams 2010, 2018; Larson 1977; Parkin 1983). While professions can be distinguished from non‐professions along multiple dimensions, some carry greater analytical weight than others. At the highest level, the distinction lies in the ability of the professions to secure a legally enforced monopoly over service provision through state‐backed licensure and legitimation (Adams 2018; Collins 1990; Freidson 1994; Larson 1977; Parkin 1983; Saks 2021).^1^ This state‐sanctioned legitimacy, in turn, provides professionals with a legal and institutional basis for monopolizing economic and symbolic resources, including power, status and authority. It also enables the professional exercise of downward power, operationalized through social closure strategies that restrict access to eligible individuals while excluding ineligibles (Parkin 1983; Saks 2015, 2021; Weber 1978). Finally, state legitimation confers a broader form of public legitimacy to professionals (Adams 2018; Freidson 1994; Parkin 1983); one that is reinforced by cultural representations of professionals and professional work in various media (e.g., film, literature, television, image and video; see Friedman 1989; Glenn 2002; Greenfield et al. 2001; Scott 2008; Starr 1982; Turow 2010). These media tend to portray professionals as—however flawed—generally competent and therefore deserving of the economic and symbolic resources they hoard (Bourdieu 1991).^2^

However, as noted above, some dimensions once considered central to professional work are gradually fading away—though not entirely disappearing. Scholars note that professional work traditionally allowed for greater worker autonomy and control—both occupational and technical—compared to non‐professional work (Adams 2018; Freidson 2001; Kalleberg 2011; Pettigrew et al. 1992).^3^ More recently, professions have undergone a turn towards standardization and financialization (Abbott 1998; Alder and Kwon 2013; Crowley et al. 2010; Faulconbridge et al. 2023; Ritzer and Walczak 1988; Timmermans and Berg 2003; Wilhelm et al. 2020), influenced partly by the rise of the New Public Management movement (Lane 2000). Nevertheless, Randall and Kindiak (2008, 345) characterize the efforts undertaken by professionals in resisting deprofessionalization as ‘postprofessionalization’. The authors charge that professionals, ‘having already attained professional status, [are] in a radically different position than a group [non‐professionals] that has not attained such status in that [they] [have] institutionalized sources of power that make it easier to resist professional decline’. For example, the authors argue that medical professionals in Ontario, Canada, have been more successful in challenging the government's authority than non‐professionals owing to the benefits of their professionalization. Notwithstanding these insights, professionals are indeed experiencing transformations in their daily experiences of work linked to shifting economic structures.

Next, I chart the macroeconomic and institutional transformations (re)shaping professional work, such as the rise of financialization (Allan et al. 2018) and the opening of employment relations (Kalleberg 2011). These transformations have altered the scenery of professional work and facilitated new forms of social closure to maintain professional advantages (see Abbott 1988; Bolton and Muzio 2007; Faulconbridge et al. 2023). For some, these transformations have made professional work more stressful and anxiety‐inducing (see Allan et al. 2018), leading me to conclude that professions are more precarious today than in the past and therefore *less* analytically distinct from non‐professions *if* we attend to earlier definitions of professional work as being non‐precarious and relatively autonomous (which I am not sure we should) (see also Saks 2021). However, if we attend to more contemporary definitions of professional work—as defined less by non‐precarity and technical control, but still by state legitimation and economic and symbolic advantages secured through social closure—then we observe that professional work remains distinct from non‐professional work. Despite some indicators of convergence (in relation to traditional definitions), I argue that professions remain analytically distinct from non‐professions because of the economic and symbolic rewards professionals continue to enjoy due to the social closure strategies they enact (granted through state legitimation) and the enduring cultural representations and ‘epistemic cultures’ they helped create to symbolically recognize, justify and naturalize their prestigious status (Scott 2008, 224; see also Bourdieu 1991; Glenn 2002; Starr 1982; Turow 2010). Hence, I argue that professions are not experiencing a ‘crisis’ but are simply under construction.

Finally, I explore how certain members of the public issue attacks on professional expertise, particularly the expertise attached to regulatory and policy science—the so‐called ‘middle‐lane’ of science (Eyal 2019). Sometimes criticism is advanced for good reason, as experts can ‘get it wrong’ and act in self‐interest (Eyal 2019). Similar to Bakan's (2020) qualms about corporate self‐regulation, there are legitimate concerns to be aired about whether expert professionals can place public interests above their own (Eyal 2019). Nevertheless, I begin by more clearly defining professions, their origins and their (mutating) characteristics.

## 2 Defining Professions

It is important to first outline how professions emerge. Professions come into being only when professional interest groups are legitimized by the state (Adams 2018; Freidson 1994; Larson 1977; Parkin 1983). Without the state's endorsement of professional privileges, such as the right to self‐regulate and control standards of entry, professions would not exist. The state grants legitimacy to professional groups because this legitimacy, in turn, affirms the state's authority and credibility (Adams 2018). Eyal (2019, 97) similarly demonstrates how professional ‘expertise’ has been increasingly mobilized by the state in recent decades to imbue its policies with legitimacy, leading to the ‘scientization of politics’, and subsequently, the ‘politicization of science’ (which spurs the attacks on expertise discussed in the final section). Hence, as Adams (2018) argues about professions and Bakan (2020) about corporations, examining the (changing) relationship between these entities and the state is paramount to understanding them.

At the outset of her first chapter exploring professions in Canadian history, Adams (2018) acknowledges that there exists no universally accepted definition of ‘professions’. Despite this admission, Adams (2018) describes the two ways professions have been defined historically and contemporarily. First, the ideal‐type, taxonomic, or trait definition was popular pre‐1970s (Greenwood 1957; Saks 2010, 2021). It argued that professionals were distinguishable from non‐professionals based on their universal attributes, including possession of arcane knowledge, advanced training or credentials, social/legal authority, and support from professional bodies and associations. Arguably, this is how many members of the Canadian public continue to view professionals today; that is, as individuals deserving of an elite status due to their expertise, rigorous education and complex work. Naturally, not all members of the public hold such sentiments, and so I endeavour to explore public attacks on professional expertise in the final section of this article. Members of the public also hold divergent views about professional groups. A 2021 Ipsos report found that Canadians rate doctors, scientists and teachers as more ‘trustworthy’ than lawyers, business leaders and politicians (IPSOS 2021). Accordingly, professions can be more or less supported, and thus face more or less blowback, depending on public perception.

Although focusing primarily on representations of race and gender, Glenn (2002) argues that cultural representation is a key mechanism through which social constructs (like professions) are imbued with meaning. Glenn (2002) contends that the deployment of symbols, rhetoric and images shapes how race and gender are understood in the public imagination. We can equally apply this framework to understand not only how the white coat becomes associated with doctors, but also the enhanced status a person wearing a white coat is granted partly due to the public's interaction with ‘professionals’ through popular culture (Friedman 1989). Greenfield et al. (2001, 4) note ‘that much “public” understanding of law is gleaned from cultural representations of the law’. For example, in their investigation into how the law and lawyers are depicted in film across the 1900s, Greenfield et al. (2001, 27) find that protagonist lawyers are regularly framed as ‘heroes’ and justice as served. These lawyers ‘become the role models to which not only lawyers but the public “should” aspire to or, rather, expect [to receive service from]’. They offer, as Starr (1982) observes, ‘cultural authority’: the ability to build reality through definitions and representations of fact. Even still, Greenfield et al. (2001, 27) note that films where justice is not served ‘do not disturb our faith in the law; the failings are those of individuals, normally police officers, who are shown as corrupt’—bad apples in an otherwise well‐oiled system.

Cultural objects, including literature, film, TV and video, combine to influence how individuals conceptualize professional work and the privileged status they do or do not attach to it. This is because these objects operate within what Bourdieu (1979, 1991) calls ‘symbolic systems’; systems that legitimize the ‘symbolic power’ of dominant social groups and their associated epistemic practices. Bourdieu envisions symbolic systems as the mechanism through which reality is constituted and communicated, whereas symbolic power captures the social forces through which the meanings the dominant group ascribes to themselves (e.g., as entitled to economic and symbolic capital because of their ‘rare’ and/or ‘valuable’ skills or ‘genius’) are naturalized and circulated for the purposes of garnering consent from the subordinate group in order to become structurally validated (see Gramsci 1971). In essence, cultural representation contributes to the legitimacy of professional work by positioning it as hierarchically superior to non‐professional work. This legitimacy, in turn, reinforces the justifications professionals use to establish and maintain social closure. As a result, both symbolic and material rewards follow from the closure of professional occupations.

The ideal‐type definition further gestures towards an earlier time when professionals were selected based on ‘patronage’ and ‘gentlemanly behaviour’, rather than what most people today would consider robust standards for entry (Adams 2018), including scoring well on examinations and training under a respected professional. However, even the ‘dispassionate’ logic attached to the rise of competency‐based standards for entry masks the continued privileging of upper‐class social and cultural capital (Bouzin 2023, cited in Beagan et al. 2024). Crucially, organizational norms and processes have yet to catch up with the changes in the demographic attributes of individuals entering professional work. While more minorities are entering professional work (Beagan et al. 2024; Bolton and Muzio 2007; Kalleberg 2011) or pursuing education to become a professional (Ballakrishnen 2023; Marom 2019), organizational barriers like the ‘ideal worker’ model that privileges a disembodied male worker (Acker 1990) continue to exert influence over organizational structures, leading to internal social closure regimes like gender segmentation (Bolton and Muzio 2007). Marom (2019) similarly argues that ‘professional microaggressions’—covert manifestations of racism and colonialism in professional organizations—emerge, in part, from a professional culture that privileges white, Westernized standards of speech, dress and comportment (see Gonin et al. 2012, on the ‘bad larder’). These standards result in Indigenous professionals being viewed as incompetent or a less ideal fit for professional work (see also Beagan et al. 2024).

Ideal‐type definitions were critiqued by scholars in the 1970s because of their universalism and failure to consider how professions vary historically, geographically and temporally (Adams 2018). However, as Marom (2019) demonstrates, these enduring stereotypes (reinforced by cultural representations, I would argue) regarding who constitutes a ‘professional’ continue to exert influence over how professional work is experienced (as welcoming, hostile, etc.) by individuals coming from various social locations. Robert and Harlan (2006) also contribute to our understanding here, as they demonstrate how disabled workers are frequently imbued with fictional identities that stress their relative deficiency (the incompetent and the helpless) compared to the able‐bodied norm. In their case, disabled workers are similarly constructed as non‐ideal types for professional work. Taken together, these studies demonstrate how the category of the professional has long been shaped by white, masculine, able‐bodied, cisgender, heterosexual and Anglo‐American norms of competence (Beagan et al. 2024). As a result, discussions of professionalism and professionalization cannot be separated from the ways these standards (re)produce and legitimize certain bodies as ‘appropriately’ professional, while positioning other bodies as deficient strictly based on their ascribed characteristics.

A second and arguably more productive way of defining professions is around the professional claim to economic and symbolic resources—the neo‐Weberian approach (Saks 2015, 2021). When professionals lay claim to social and legal authority, they necessarily create a relational category (Collins and Bilge 2016; Glenn 2002) between themselves and a ‘non‐professional’ class of workers. Wright's (2005) ‘inverse interdependent welfare’ and ‘opportunity hoarding’ principles are useful to consider here. The inverse interdependent welfare principle stipulates that the welfare of one group (professionals) necessarily depends on the ill‐fare of another group (non‐professionals). The opportunity hoarding (or exclusion principle) further states that one group (professionals) can only gain relative welfare over the other group (non‐professionals) when they exclude the latter from access to valuable opportunities, resources and socioeconomic rewards.

Social closure is the primary mechanism through which professionals guard their special resources (Larson 1977; Parkin 1983; Weber 1978). Social closure strategies can be external (preventing individuals from entering the profession) and internal (preventing the upward mobility of certain individuals or social groups once they gain entry). External social closure, or ‘gatekeeping’, typically involves professional associations determining the credentials required to gain access to a profession (e.g., a PhD to access tenure‐track jobs in academia). Using the power‐threat hypothesis, Chamberlain et al. (2008) argue that sexual harassment functions as an internal social closure mechanism that serves to punish role‐deviant women and preserve men's monopoly over valuable resources within organizations. Bolton and Muzio (2007) similarly document the internal gender segmentation regimes in the legal profession that exclude women from senior positions, relegate women into feminized legal specializations, and encourage women to internalize feminine stereotypes. They further elucidate how feminized specializations receive less pay, are associated with fewer chances for career progression, and are undervalued relative to masculinized specializations.

Similarly, Galabuzi (2010) postulates that racial discrimination operates as a social closure mechanism through which racial majorities seek to maintain their labour market privileges through the subordination of racial minorities. Beagan et al. (2024, 241) demonstrate that some of the strategies used to subordinate marginalized professionals include promulgating ‘hostile misperceptions’, implying or outright asserting that marginalized professionals are less qualified for their jobs (and subsequently ‘stole’ them from ‘more deserving’ candidates), and had an unfair advantage in acquiring their jobs (due to affirmative action or employment equity policies). These findings indicate that the historical gentleman's club (see Adams 2018) of the (legal) profession is maintained even as the social groups not historically included in the profession gain access to it. These findings indicate broadly that while the professions are opening up externally (Ballakrishnen 2023), they are clamping down internally (Beagan et al. 2024; Bolton and Muzio 2007; Faulconbridge et al. 2023).

Another traditionally important dimension of professional work is the relative autonomy and occupational and technical control professionals have over the labour process compared to non‐professionals (Abbott 1988; Adams 2010, 2018; Larson 1977; Pettigrew et al. 1992). Professional associations set the standards for how an entire field should conduct its work. In restricted title professions (e.g., accounting), anyone can practice, but only those who are registered or licensed can adopt the professional title and reap its associated benefits (Adams 2018). Yet, even if someone doing accounting work is not a licensed accountant, how they conduct their work is influenced, if not governed, by the standards set by professionals, giving the latter power over the former. Meanwhile, although professionals must abide by certain standards and rules, the process one takes in their work can differ. For example, while a professor is provided with benchmarks for what a course must entail, the readings, lecture style and evaluation components are up to their discretion. Some choose to assign one major essay, while others get students to answer weekly discussion questions; some teach with dozens of slides, while others come prepared with activities. Non‐professionals often lack such creative and personality‐inflected luxuries. Instead, their jobs have less room for variation. Take the contemporary cashier as an example. Their job demands that they scan the products that move towards them on a conveyor belt and charge the customer the amount that appears on the cash register's screen. Some stores also demand they issue the ‘correct’ greeting and farewell (see Hochschild 1983, on Winn‐Dixie policies), making the entire process standardized.

Marxist political economist Braverman (1974) argues that standardization (which he argues leads to ‘deskilling’) is the product of scientific management. Scientific management refers to management's use of scientific methods to more effectively control the labour process and increase productivity (Braverman 1974). It is undertaken to eliminate time‐wasting and individually nuanced processes to advance capital interests by increasing the surplus value extracted from workers. Scientific management involves breaking the labour process down into smaller component parts and standardizing how each labour task is performed. For example, when I worked at a popular burger chain as a teenager, each worker was responsible for one part of the labour process. Some workers were assigned to the ‘front’, taking orders and inputting them into the technology that sends them to ‘kitchen workers’ to prepare. Meanwhile, the kitchen workers were responsible for preparing the orders (in the mandated fashion) and sending them back (through a chute [machine]) to ‘front staff’ to deliver to customers. This division of labour is enacted by management because it gets the order to the customer quickest. The result is happier customers who are more likely to come back and spend their dollars, which go into shareholders’ pockets after paying out workers a subsistence wage that does not reflect the total effort they contributed to the labour process (Marx 1976, cited in Cole et al. 2022).

Braverman (1974) has been criticized by neo‐Weberians for being too monolithic and deterministic in his account, glossing over worker agency and resistance, neglecting occupational stratification and status differences, and overlooking how capitalism can also lead to upskilling, particularly among professionals (Saks 2021). Importantly, he fails to consider the key tenet of the postprofessionalization thesis (Randall and Kindiak 2008): that professions, having already attained professional status, are in a stronger position to resist standardization than non‐professional occupations given their relationship to institutional power.

With that said, research does suggest that various macroeconomic changes affecting the professions have made it so this standardization (what Ritzer [1993], cited in Pettersen (2018 refers to as McDonaldization given the emphasis on efficiency, calculability, predictability and control) is no longer strictly observed in non‐professional work (Crowley et al. 2010; Kalleberg 2011; Ritzer and Walczak 1988). Increasingly, professionals are facing pressures around standardization, work intensification and performance metrics, leading some scholars to wonder if professions remain analytically distinct from non‐professions. These transformations have been influenced partly by the New Public Management movement, which sought to reform the public sector to more closely mirror the ‘efficient’ practices associated with the private sector (Lane 2000). The result has been increased standardization, competition and managerialism influencing professional work. What stands out to me in these discussions is the increasing levels of precariousness professionals report, especially the law partners in Allan et al.'s (2018) study. Precariousness has traditionally been associated with non‐professional work (Kalleberg 2011), but it appears the tide is changing on that front.

## 3 Precariousness in Professional Work

Dahlin (2024, 4) defines precarious employment as ‘employment that is uncertain because of a lack of opportunity, skill, or education’. Vosko (2006) argues that precarious employment refers to work that is low‐waged and insecure, has limited fringe benefits, and puts the worker at a high risk of health problems. Shuey et al. (2018) usefully add that precarious work, which they define as insecure, non‐standard and non‐permanent jobs, stems from the decimation of long‐term employment relations—something Kalleberg (2011) also observes. However, Alberti et al. (2018) importantly note that precarity has become a diluted and muddied concept. They argue that precarity should be understood as a *process* that takes shape through objective social conditions *and* subjective experiences of one's employment rather than a static quality attached to one class of workers. Their conceptualization is important because it insists that precariousness is not a social class indicator but rather a social relation that *all* workers experience under capitalism. Bakan (2020) echoes this sentiment when he observes that most people's lives are becoming more precarious due to stagnating wages, defunded public services, the evaporation of ‘good’ jobs, and the growth of inequality between classes under capitalist regimes (see McCall and Percheski 2010, for more on the latter). Acknowledging this, Kalleberg (2008) argues for the need to re‐emphasize precariousness in academic and public inquiry and to focus on the *employment relations* that facilitate it, as these relations impact both professional and non‐professional work. While it is surely empirically founded that some social classes are more likely to experience precariousness than others (see Kalleberg 2011; Vosko 2006), it would be fallacious to claim that members of the privileged social classes (however defined) or professions do not experience at least the *subjective* feelings connected to precariousness. Alberti et al. (2018, 449) explain that subjective feelings of precarity may include insecurity, vulnerability, and ‘the loss of grip over a future that once seemed under control’.

In an article aptly titled ‘The fearful and anxious professional’, Allan et al. (2018) interviewed law partners and came away with a finding that contradicts hegemonic cultural representations of the professional lawyer (Friedman 1989): that law partnership, once considered the secure and stable end of one's career, is now a status to be repeatedly worked for and (re)achieved or lost. The professionals interviewed expressed *fear* and *anxiety* over their ability to retain their privileged status. Specifically, they expressed fear of failure and being cast out or otherwise regarded as inferior by the in‐group. They also demonstrated anxiety about the future of their careers because of the increasing performance expectations dictated by their firm.

Crucially, Allan et al. (2018) connect this transformation in law partnership to the rise of financialization regimes (enabled by New Public Management ideas and practices) that emphasize performance metrics. Under financialization, employees are valued not for their collegiality, expertise or time spent with the firm (i.e., traditional metrics); they are valued strictly for their financial contributions. Kalleberg (2008) has also identified neoliberal globalization, union decline and the withdrawal of the welfare state as key mechanisms through which precariousness has grown in the United States. Allan et al. (2018), citing Costa and Grey (2014), note that individuals accept and participate in disciplinary regimes with the hope that their imaginary future selves will manage to escape or otherwise sit at the top of the regime. Yet, Allan et al.'s (2018) law partners *are* (arguably) at the top of the regime, and yet precariousness still finds root inside them. This recognition led Allan et al. (2018, 125) to conclude that ‘[financialization] encourages practices that contradict the logic of professional partnerships and [*sic*] values’, indicating the values that once marked the professions are changing.

## 4 Is Professional Work Disappearing?

The question soon becomes: if professions are traditionally distinctive from non‐professions via their values and autonomy, and if financialization, neoliberalization, rationalization and other macroeconomic transformations linked to New Public Management have rendered the professions more precarious, do they still remain analytically distinct from non‐professions? The answer, I believe, is yes. If the reader can recall the earlier definition of the professions as constructs that are historically, geographically, and temporally amenable, it stands that professions are not static entities but are constantly evolving amid macroeconomic transformations. In conclusion, we can deduce that transformations are definitionally congruent with professional work.

Adams’ (2018) work is crucial in making this point clear, as she demonstrates how professions have changed in pre‐ and post‐Confederation Canada. In her analysis exploring professions prior to the 1900s, Adams (2018) highlights that ‘the powers conferred upon professional regulatory bodies *varied widely* across profession, time, and province’ (p. 84, emphasis added). ‘[Yet] at the bare minimum’, she notes, ‘bodies were granted the right to oversee entry to practice (or access to a restricted title)’ (i.e., occupational control) (p. 84). These principles tell us that while some characteristics are enduring (i.e., overseeing entry and title access—attributes secured through state legitimation), others may rise and fall in prominence; for instance, those that select professionals based on elusive qualities and patronage or those that connect professional work with technical control.^4^ Moreover, Adams (2018) demonstrates that professional regulation was highly debated in the legislature and public discourse. Between 1868 and 1900, 346 bills were introduced in Canadian legislatures to expand *and* restrict professional self‐regulation (Adams 2018). These numbers tell us that professions have long been sites of debate and power consolidation (Larson 1977), with some parties hoping to expand professional self‐regulation, others wanting to access it for themselves, and still others seeking to restrict it.

Abbott (1988) complements this line of thinking by demonstrating how technological advancements reshape how professional work is performed and enable claims to be made by new groups vying for professional status. For example, transformations in medical technologies and the resulting standardization of diagnostic work have enabled technicians and radiologists to make stronger claims to expertise and authority. If they can be trained to interpret medical images or perform tasks traditionally reserved for professionals, they may argue that they can perform them equally well and therefore deserve access to the title and benefits of professional status. Yet, as Faulconbridge et al. (2023) demonstrate, professionals respond to technological advancements (in their case, artificial intelligence [AI]) by engaging in intertwined boundary work to redefine and create new boundaries that restrict incursions into their work and protect their symbolic and material interests. Although Faulconbridge et al.’s (2023) lawyers brought technologists (non‐professionals) into the professional fold as a response to technological advancements, they reinstated themselves as hierarchically superior through their negotiated claim that while AI can accomplish the basic functions traditionally associated with their work, it cannot produce the essential, sophisticated tasks that only ‘real’ lawyers can conduct on the basis of their tacit professional expertise (see Eyal 2019, for a more detailed analysis). This view is supported by Pettersen (2018), who argues that because (process) knowledge work is highly complex, social, relational, and contextual, it is not easily replaceable by AI. What this means for professions rooted in process and abstraction is that they ‘can redefine [their occupation's] problems and tasks, defend them from interlopers and seize new problems’ (Abbott 1988, 9), just as Faulconbridge et al.’s (2023) lawyers did in response to the entanglement of law and AI technologies. This ability, Abbott (1988, 9) argues, is what uniquely ‘enables survival in the competitive system of professions’ versus non‐professions. Eyal (2019, 31) further insists that the novel technologies professionals use do not cause ‘deskilling, but redistribution of expertise’, which continues to make professional work relatively distinctive.

## 5 Attacks on Professional Expertise

I have demonstrated that, although variable across social contexts, professional work historically and contemporarily confers greater economic and symbolic resources upon professionals compared to non‐professionals. These privileges persist because professionals respond to macroeconomic transformations by exercising new forms of social closure (i.e., boundary work) designed to restrict incursions. Their power lies in their ability to define the norms and standards governing their fields. Despite increasing precariousness within the professions, professionals continue to engage in more complex, social and skilled forms of work. Despite the challenges to expertise that this section will briefly address, the cultural representation of the professional remains one associated with more symbolic power than the non‐professional. Regardless of the material condition of professions, the cultural/symbolic framing of professional work continues to play a key role in shaping public perceptions of its value and distinctiveness (Friedman 1989; Glenn 2002; Greenfield et al. 2001; Scott 2008; Starr 1982; Turow 2010).

Before closing this article, I would like to spotlight Eyal's (2019, 3) recognition ‘that we are in the midst of an all‐out assault on expertise’. Not all experts are professionals, but many professionals are experts, so I would like to engage this phenomenon briefly. A potential argument against continued professional distinctiveness is that, if professions are defined according to the status the public places upon them, and if this exclusive status dwindles, then professions fail to remain analytically distinct from non‐professions. However, as Hofstadter (1963, cited in Eyal 2019, 4) notes in rebuttal, anti‐intellectualism is ‘a manifestation not of a decline in [the intellectual's] position but of his increasing prominence’. Put differently, the pushback the professional expert faces does not mean their status is weakened but rather strengthened; that is, backlash signifies the prominence accorded to expertise in contemporary societies. It illuminates the lack of control ordinary people feel over their increasingly precarious lives in a society ‘of unprecedented reliance on science and expertise’ (Eyal 2019, 4). Eyal (2019) contends that such feelings often fuel a resentment towards those perceived as possessing the knowledge (or, more importantly, Eyal [2019] notes, the ‘social recognition’ [p. 30]) that helps formulate the policies that govern their lives. Partly then, being suspicious of experts is a way of enacting revenge on the expert and assuming a greater (but false) sense of control over one's life. Accordingly, such resentment indicates the continued elevated and hierarchical position of the professional, leading me to conclude that professional work remains analytically distinct from non‐professional work despite the transformations it is currently undergoing.

These remarks should not be interpreted as suggesting that all members of the public attack or distrust expertise, nor that all forms of expertise are equally criticized. Eyal (2019, 7) importantly cautions against a moral panic about a generalized assault on science, arguing that ‘not all of “science” is under assault’. It is the ‘science’ in the middle‐lane of his highway metaphor—regulatory and policy science—that has been subject to the greatest scrutiny. This form of science is most vulnerable to criticism because it can be less rigorous, hurried through production, susceptible to biased funding from industries and corporations, and hard to amend after its policy implementation. Eyal (2019) also cites research demonstrating that individuals are more likely to trust academic scientists than those working at or affiliated with private corporations and that people tend to be more mistrusting of specific topics (e.g., vaccines) as opposed to ‘science’ broadly. Hence, the ‘type’ of science and expertise under scrutiny matters, as it reveals that public distrust is not indiscriminate but instead reflects concerns about how expertise is produced, applied, and institutionalized within decision‐making contexts.

It is also important to consider which social groups are more or less trusting of expertise and why this is the case. Studies show that racial and ethnic minorities, particularly non‐Hispanic Black individuals, as well as politically conservative populations, are more likely to exhibit vaccine hesitancy, defined as reluctance to be vaccinated, than their counterparts (Bagasra et al. 2021; Zhang et al. 2023). However, the mechanisms driving vaccine hesitancy differ between these groups and reflect distinct historical experiences, levels of institutional trust and susceptibility to misinformation. For instance, vaccine hesitancy among non‐Hispanic Black populations in North America can be partly attributed to contemporary and historical sources of medical injustice. These include being more likely to report experiencing racial discrimination when trying to access healthcare services, Black women experiencing greater rates of forced sterilization than white women, and the legacy of historical injustices such as the Tuskegee Syphilis Study, in which Black men with syphilis were deliberately left untreated to study the disease's progression, even after effective treatment became available.

Conversely, politically conservative populations tend to exhibit greater vaccine hesitancy than liberals, partly because they are more susceptible to misinformation, which shapes levels of trust in scientific experts, government, and social institutions and can, in turn, contribute to vaccine hesitancy (Roozenbeek et al. 2020). For these reasons, it is important to reiterate that ‘attacks’ on science are not uniformly expressed across populations, nor are they directed at ‘science’ as a whole. Instead, attacks on science often stem from historical and ongoing injustices, the spread of and vulnerability to misinformation, and a broader sense of diminished control over one's life outcomes. As this article suggests, rising precariousness (objectively and subjectively) across the socioeconomic spectrum is likely to influence perceptions of professional experts and the contestation they encounter. Yet, equipped with their toolkit of legitimation strategies, experts are likely to regard this as little more than a temporary disruption.

## 6 Concluding Remarks

This article engaged with scholarship on work, occupations, and professions to address ongoing debates about whether professional work remains analytically distinct from non‐professional work. These debates have intensified in recent decades, particularly as professional work in the Anglo‐American context has become increasingly subject to the rationalization and standardization historically associated with non‐professional work.

Guided by the work of Adams (2018), Saks (2021) and their associated neo‐Weberian approaches, I argue that because professions are socially constructed and historically, geographically and temporally contingent, a universal definition of professions is untenable. Instead of relying on a fixed definition, it is more accurate to distinguish between features that remain relatively stable and central—especially state legitimation, which underpins occupational monopoly, control and closure—and those that are historically contingent and liable to erode over time. Thus, the weakening of historically defining features of professional work, such as relative autonomy and technical control, can coexist with ongoing professional distinctiveness.

At the same time, Goldthorpe (2002) reminds us that while ‘good jobs’ have undergone degradation since the 1970s (and are thus, in certain respects, ‘*less good*’ than they used to be), they are hardly indistinguishable from ‘bad jobs’. Put differently, while professions have been exposed to greater rationalization and subjective forms of precariousness in the last 50 years, it would be irresponsible to claim that these jobs do not remain far more advantageous than those occupied by non‐professional workers, particularly in terms of symbolic and economic rewards. Indeed, most professional, administrative and managerial workers continue to occupy long‐term, stable and well‐remunerated jobs accompanied by significant fringe benefits. Hence, while this article demonstrates the rise of precariousness within professional work, it should be noted that professional work remains a site of advantage and public aspiration (see Lehmann 2019).

Finally, this article cautions against assuming that anti‐intellectualism or public disdain towards professionals is a precursor towards attacks on their status, power and authority that will culminate in professional work losing its analytical distinctiveness. Drawing on Eyal (2019) allows me to demonstrate how the ‘anti‐expert’ tide we are currently wading through in the Anglo‐American context is the latest iteration of a recursive cycle that we have seen before. By resisting the temptation of the fallacy of discontinuity, we can recognize that anti‐expert sentiment is not a fundamentally new phenomenon. In fact, rather than signalling the demise of the professional expert, these sentiments illustrate the expert's continued relevance in a socio‐historical period where expertise shapes how we live our private and communal lives.

The article concludes that, despite ongoing restructuring driven by changing economic structures and technological advancements, professions remain distinct from non‐professions due to their continued state‐backed legitimacy and repertoire of social closure strategies, suggesting that, even in a changing world, professionals will not concede their advantages anytime soon.

## Funding

The author has received no financial support for the research, authorship and/or publication of this article.

## Conflicts of Interest

The author declares no conflicts of interest.

## Endnotes

1 Some scholars have suggested that professions’ state legitimation is increasingly under threat. However, Adams (2018) demonstrates that state intervention is not a fundamentally new phenomenon in the Canadian case, but rather a long‐standing feature of professional regulation. Similarly, Eyal (2019) argues that the state depends on professions to help constitute and validate its own authority, rather than simply constraining them. Moreover, while non‐professionals are increasingly included in regulatory and advisory bodies (e.g., medical boards), they remain a minority within these arrangements (Saks 2021). Even where lay representation is formally present, its substantive influence may be limited. Saks notes: ‘Even when lay people have representation, moreover, they are frequently ill‐equipped to make expert comment on technical issues and may only be involved in setting up, not implementing, professional project. Their experience may also be used to legitimate professional decisions already made—rather than acting as a corrective’ (p. 89). While these activities have ‘helped to limit the autonomous professional power once held before the 1960s and 1970s’ (p. 89), state legitimation remains a salient feature.

2 Professionals who act unethically tend to be framed as ‘bad apples’. At times, a professional organization is framed as a ‘bad barrel’. However, there is rarely an interrogation of the broader context (‘the larder’) a professional organization or member occupies. See Michael Gonin et al. (2012, 31), who argue ‘that the first step toward ‘healthier’ apples and barrels is to sanitize the larder’—to develop a professional climate that promotes ‘apple’ and ‘barrel’ ethicality.

3 Professionals continue to enjoy greater autonomy, occupational control, and technical control than non‐professionals; however, the rise of financialization and rationalization in the 1970s–1980s has rendered professional work increasingly precarious.

4 Feminist, antiracist, decolonial and disability scholarship highlights that the able‐bodied, white, Westernized ‘gentleman's club’ of professional work continues to pose barriers to the integration, acceptance, and recognition of marginalized groups within professional spaces. This scholarship also shows that researchers must attend to phenomena beyond hiring, as career progression, wage increases, etc., are where discrimination remains particularly manifest.
